# Prevalence and associated factors of diabetes mellitus among tuberculosis patients in Hanoi, Vietnam

**DOI:** 10.1186/s12879-018-3519-5

**Published:** 2018-11-29

**Authors:** N. B. Hoa, P. D. Phuc, N. T. Hien, V. Q. Hoa, P. H. Thuong, P. T. Anh, N. V. Nhung

**Affiliations:** 1 Vietnam National Lung Hospital, National Tuberculosis Programme Vietnam, 463 Hoang Hoa Tham street, Badinh District, Hanoi, Vietnam; 2 Centre for Operational Research, International Union Against Tuberculosis and Lung Disease, Paris, France; 3 Center for Public Health and Ecosystem Research, Hanoi University of Public Health, Hanoi, Vietnam; 4 Institute of Environmental Health and Sustainable Development, Hanoi, Vietnam; 5 Hanoi Lung Hospital, Hanoi, Vietnam; 6 Vietnam Association for Tuberculosis and Lung Disease, Hanoi, Vietnam

**Keywords:** Tuberculosis (TB), diabetes mellitus (DM), prevalence, Risk factors, Vietnam

## Abstract

**Background:**

Diabetes mellitus (DM) is recognized as an important comorbidity for the development of tuberculosis (TB). With the increase of DM burden globally, concerns have been raised about the emerging co-epidemics of DM and TB, especially in low- and middle-income countries.

**Methods:**

A facility-based, cross-sectional study was carried out in all 30 district TB units in Hanoi, Vietnam. All eligible, diagnosed TB patients aged 15 years old or older were asked to provide consent and were screened for diabetes using fasting blood glucose (FBG). Pre-tested semi-structured questionnaires were used for collecting demographic data, lifestyle habits and clinical data. Identification of pre-diabetes or diabetes in TB patients was done in accordance to parameters set by the American Diabetes Association (2016).

**Results:**

Of 870 eligible TB patients, 831 (95.5%) participated in the study. Of those, 241 (29%; 95%CI: 25.9–32.1%) were prediabetic and 114 (13.7%; 95%CI: 11.4–16.1%) were found to have DM. The risk of DM was higher in patients belonging to the age group 40–64 years (OR 6.09; 95%CI 2.81–13.2); or the age group 65 years or older (OR 2.65; 95%CI 1.65–4.25) or who have a family history of DM (OR 2.71; 95%CI 1.33–5.50).

**Conclusions:**

This study demonstrated high prevalence of DM and prediabetes among TB patients in Hanoi, Vietnam. National Tuberculosis Programme needs to establish a systematic screening process for DM among TB patients.

## Background

Vietnam is a lower middle-income country and ranks 15th amongst the 30 highest Tuberculosis (TB) incidences globally [1]. The World Health Organization (WHO) estimated that there were 126,000 TB incidence cases in Viet Nam in 2016, which accounts for 123 cases per 100,000 population. The number of reported TB cases in Vietnam in 2016 was 106,527 TB cases [1]. The burden of Diabetes mellitus (DM) is increasing worldwide with the International Diabetes Federation (IDF) estimating that approxiately 425 million people are living with diabetes, amounting to 8.8% of the global population [2]. It was also estimated that approximately half of this population was unaware of their DM status in 2016 [2]. Low- and middle-income countries account for approximately 80% of the global DM burden, and more than 90% of the global TB burden [2].

DM is recognized as an important comorbidity for the development of TB, with those who have DM having a two to three times higher risk of developing TB than those without DM [3–5]. This leads to a higher prevalence rate of TB among DM patients; the rate of DM is also higher among TB patients than in general population [3]. People with TB and DM generally have a poorer response to TB treatments and increased the risk of relapse and death [3, 6, 7]. With the increase of DM burden globally, concerns have been raised about the emerging co-epidemics of DM and TB, especially in low- and middle-income countries.

In 2011, the WHO and the International Union Against Tuberculosis and Lung Disease (the UNION) recommended that TB patients should be routinely screened for DM [3]. The framework recommended all countries should be surveilling diabetes among TB patients [3]. Many studies conducted in different countries have shown the prevalence of DM among TB patients to be approximately 12–44% [8]. Currently, in Vietnam, there is no systematic screening for DM amongst TB patients and limited studies investigating the prevalence of DM amongst TB patients. One study conducted in the National Lung Hospital in 2006–2008 found the prevalence of DM among TB patients to be 8.8% [9].

Hanoi is the capital of Vietnam, has a high proportion of the total TB cases reported in the country. In 2016, Hanoi detected and reported 4382 TB cases, amounting to 59 cases per 100,000 population. This study aimed to identify the prevalence and some associated risk factors of DM amongst TB patients attending district TB units in Hanoi, Vietnam. The results will look to help to establish linkages between TB and DM and provide data to estimate the needs and requirements for DM care services among TB patients in Vietnam.

## Methods

### Setting and study population

This facility-based, cross-sectional study was conducted in all 30 districts of Hanoi where the total population was estimated at 7,328,400 in 2016. The study population included all TB patients aged over 15 years old, who had been diagnosed and enrolled for TB treatment from October to December of 2016.

The sample size was calculated based on the standard formula for estimating a single population:

$$ n=\frac{Z_{1-\alpha /2}^2\cdot p\cdot \left(1-p\right)}{d^2}\times DE. $$

A previous study conducted in 2006–2008, at the National Lung Hospital in Hanoi, investigated the DM prevalence among 2867 newly diagnosed TB patients and discovered the DM prevalence to be 8.8% [9]. A similar DM prevalence was assumed for this study, with a 95% confidence interval and 20% relative precision. With a cluster sampling of 1.5 the sample size was calculated to be 740 participants. To account for an estimated non-participation rate of 15%, the final sample size required for this study was considered as 870 TB patients.

### Sampling method

Patients who were aged 15 years or above and had been diagnosed with TB in any 30 districts of Hanoi were considered for the study. Participants were enrolled for TB treatment between October and December of 2016. Recruitment was continuous until the required sample size was met.

### Data collection

All consenting participants were interviewed using pre-tested questionnaires to collect the patient’s information. Questionnaires collected socio-demographic variables including age, sex, weight, height, education, occupation, marital status, average monthly income and HIV status. The questionnaires also collected information around lifestyle factors including smoking, drinking and exercise. The participant’s disease profile and clinical records pertaining to TB and DM status were also collected. TB patients were asked about their DM status. If the TB patients were diagnosed with DM prior to the study, they were not further investigated for the DM, and the patients were requested to provide their most recent DM test results. If the TB patients were did not know or were unsure about their DM status, they were requested to undergo a fasting blood glucose (FBG) test, after the cessation of eating for at least 8 h.

### Definition

TB patients’ diagnosis and treatment outcomes were categorized following NTP’s guidelines, which are in line with WHO’s recommendations [10].

Patients with FBG levels ≥126 mg/dl (~ 7 mmol/l) were diagnosed as having DM. Patients with FBG levels between 100 and 126 mg/dl (5.6–6.9 mmol/l) were diagnosed as having impaired fasting glucose (IFG), or prediabetes, in line with guidance from the American Diabetes Association (ADA) [11].

Classification of socioeconomic status was determined following Vietnam Government criteria, issued by Prime Minister for the period 2016–2020 [12]. A “poor” household is one whose per capita income does not exceed 700,000 Vietnam Dong (VND) in rural area and 900,000 VND in urban area. Households are also classified as poor if their per capita income is between 700,000 – 1,000,000 VND in rural areas or 900,000-1,300,000 VND in urban areas when households have unfavourable outcomes in at least three of ten basic social service indicators (access to medical services, health insurance, education level of adults, school attendance of children, housing quality, average housing area per capital, residential water sources, hygienic latrines and toilets, telecom services, and assets to serve information access (TV, computer, radio, other). A “near-poor” household is one whose per capita income is between 700,000–1,000,000 VND or 900,000-1,300,000 VND in rural or urban areas, respectively, and have unfavourable outcomes in at least three of ten basic social service indicators A “medium” household is a household whose per capital income is between 1,000,000–1,500,000 VND in rural area and between 1,300,000-1,950,000 VND in urban area [12].

### Statistical analysis

Data was entered into EpiData version 3.1 (EpiData Association, Odense, Denmark) and the analysis were carried out using Stata v.13 software (Stata Corporation, College Station, TX, USA). The main outcomes of the analysis were the number and proportion of TB patients with DM and prediabetes; data was then stratified by sex, age, education, occupation, marital status, residence, monthly incomes, socio-economic status and body mass index (BMI). The proportion of TB patients with DM and prediabetes was also stratified by lifestyle and other factors such as physical activity, smoking, drinking status, family history of DM, type of TB, treatment category and HIV status. Single proportion with 95% confidence interval (CI) was calculated. A Kruskal-Wallis test and Cuzick test were used for the nonparametric data and to test for trend across ordered groups. Odds ratio (OR) with 95% CI were used to describe association between groups. Multivariate logistic regression analysis was performed to calculate adjusted OR for analyzing the association of the related risk factors with the outcome variables.

### Ethics approval

The study protocol was approved by the Institutional Review Board of the National Lung Hospital, Hanoi, Vietnam. Patients diagnosed with DM received appropriate treatment. Written informed consent was obtained from each participant prior to enrolment.

## Results

A total of 851 eligible TB patients aged ≥15 years old were enrolled during the study period. 831 (97.7%) patients consented and were interviewed.

### General characteristics

The socio-demographic characteristics of study’s participants are presented in Table 1. Of 831 TB patients, 549 (66.1%) were males and 282 (33.9%) were females. The mean age of participants was 48.4 (standard deviation (SD): 18.2). More than half of the participants (531, 63.9%) had a secondary or high school education. The number of participants residing in urban areas was 401 (48.3%) and 468 (56.3%) had a monthly income less than 3,000,000 VND. The mean of BMI was 19.6 kg/m^2^, (SD 3.0) with 34.8% patients was classified as underweight.

**Table 1:** General characteristics of Tuberculosis patients enrolled for the study, Hanoi, Vietnam, 2016. (*n* = 831)

General characteristic	n	%
Total	831	*100.0*
Sex		
*Male*	549	*66.1*
*Female*	282	*33.9*
Age in years		
*15–24*	92	*11.1*
*25–34*	133	*16.0*
*35–44*	142	*17.1*
*45–54*	137	*16.5*
*55–64*	175	*21.1*
*65 years and older*	152	*18.3*
*Mean age (SD)*	48.4 *(18.2)*	
Education		
*Primary and lower*	143	*17.2*
*Secondary; high school*	531	*63.9*
*Colleagues/University or higher*	157	*18.9*
Occupation		
*Unemployed*	113	*13.6*
*Farmer*	259	*31.2*
*Self-employed*	49	*5.9*
*Government employed/ Student; pupil / retired*	410	*49.3*
Marital status		
*Single*	158	*19.0*
*Married*	629	*75.7*
*Divorced / separated*	44	*5.3*
Residence		
*Rural*	430	*51.7*
*Urban*	401	*48.3*
Monthly Income (VND)		
*< 3,000,000 (~ 132.3 US$)*	468	*56.3*
*3,000,000 - 5,000,000 (~ 132.3–220.5 US$)*	220	*26.5*
*> 5,000,000 (~ 220.5 US$)*	143	*17.2*
Socio-economic status		
*Poor/ near-poor household*	103	*12.4*
*Medium household and above*	728	*87.6*
BMI, kg/m2		
*Underweight, < 18.5*	289	*34.8*
*Normal, 18.5–24.9*	518	*62.3*
*Overweight, > = 25*	24	*2.9*
*Mean (SD)*	19.6 *(3.0)*	

*SD Standard deviation*; VND* Vietnam dong*; BMI* Body mass index

### Prevalence of prediabetes and DM among TB patients

The overall prevalence of prediabetes and DM among TB patients and stratified by general characteristics are shown in Table 2. Of 831 TB patients, 241 (29%; 95% CI: 25.9–32.1%) were prediabetic and 114 (13.7%; 95% CI: 11.4–16.1%)) were found to have DM. The prevalence of DM was higher among males than females (16.0% vs 9.2%) and was shown to increase with age. The incidence of prediabetes was also higher among those in older age groups, those who had primary or lower education levels, those who worked as farmers and in those with a monthly income less than 3,000,000 VND.

**Table 2:** The prevalence of pre-diabetes and DM among TB patients, in Hanoi, Vietnam, 2016 (*n* = 831)

	Total TB patients evaluated for DM (n)	TB patients with pre-DM (n)	Prevalence of pre-DM (%; 95% CI)	*p* value*	TB patients with DM (n)	Prevalence of DM (%; 95% CI)	*p* value*
Total	**831**	**241**	**29.0 (25.9–32.1)**		**114**	**13.7 (11.4–16.1)**	
Sex				0.274			0.007
*Male*	549	166	30.2 (26.4–34.1)		88	16.0 (13.0–19.1)	
*Female*	282	75	26.6 (21.4–31.8)		26	9.2 (5.8–12.6)	
Age, years				**< 0.001**			**< 0.001**
*15–24*	92	19	20.7 (12.2–29.1)		5	5.4 (0.7–10.1)	
*25–34*	133	23	17.3 (10.8–23.8)		6	4.5 (0.9–8.1)	
*35–44*	142	37	26.1 (18.7–33.4)		16	11.3 (6.0–16.5)	
*45–54*	137	47	34.3 (26.3–42.4)		27	19.7 (13.0–26.5)	
*55–64*	175	61	34.9 (27.7–42.0)		33	18.9 (13.0–24.7)	
*≥ 65*	152	54	35.5 (27.8–43.2)		27	17.8 (11.6–23.9)	
Education				**0.002**			0.624
*Primary or lower*	143	49	34.3 (26.4–42.1)		19	13.3 (7.7–18.9)	
*Secondary; high school*	531	163	30.7 (26.8–34.6)		77	14.5 (11.5–17.5)	
*Colleagues/University or higher*	157	29	18.5 (12.3–24.6)		18	11.5 (6.4–16.5)	
Occupation				**0.002**			0.328
*Unemployed*	113	35	31.0 (22.3–39.6)		15	13.3 (6.9–19.6)	
*Farmer*	259	96	37.1 (31.1–43.0)		28	10.8 (7.0–14.6)	
*Self-employed*	49	15	30.6 (17.2–44.0)		9	18.4 (7.1–29.6)	
*Government employed/ Student; pupil / retired*	410	95	23.2 (19.1–27.3)		62	15.1 (11.6–18.6)	
Marital status				0.062			0.481
*Single*	158	34	21.5 (15.0–28.0)		17	10.8 (5.9–15.6)	
*Married*	629	192	30.5 (26.9–34.1)		91	14.5 (11.7–17.2)	
*Divorced/separated*	44	15	34.1 (19.5–48.7)		6	13.6 (3.1–24.2)	
Residence				0.084			0.107
*Rural*	430	136	31.6 (27.2–36.0)		51	11.9 (8.8–14.9)	
*Urban*	401	105	26.2 (21.9–30.5)		63	15.7 (12.1–19.3)	
Monthly income (VND)				**< 0.001**			0.636
*< 3,000,000 (~ 132.3 US$)*	468	153	32.7 (28.4–37.0)		60	12.8 (9.8–15.9)	
*3,000,000 - 5,000,000 (~ 132.3–220.5 US$)*	220	65	29.5 (23.5–35.6)		35	15.9 (11.0–20.8)	
*> 5,000,000 (~ 220.5 US$)*	143	23	16.1 (10.0–22.2)		19	13.3 (7.7–18.9)	
Socio-economic status				0.794			0.790
*Poor/ near-poor household*	103	31	30.1 (21.1–39.1)		15	14.6 (7.6–21.5)	
*Medium household and above*	728	210	28.8 (25.5–32.1)		99	13.6 (11.1–16.1)	
BMI, kg/m2				0.077			0.304
*Underweight, < 18.5*	289	94	32.5 (27.1–38.0)		38	13.1 (9.2–17.1)	
*Normal, 18.5–24.9*	518	142	27.4 (23.6–31.3)		69	13.3 (10.4–16.3)	
*Overweight, > = 25*	24	5	20.8 (3.3–38.4)		7	29.2 (9.6–48.8)	

*TB* Tuberculosis*; DM* Diabetes mellitus*; CI* Confidence interval*; SD* Standard deviation*; VND* Vietnam dong*; BMI* Body mass index

*p value by Kruskal-Wallis test and Cuzick test were used for the nonparametric data and to test for trend across ordered groups

### Lifestyle factors

The proportion of DM among TB patients with a family history of DM was 29% (95% CI: 18.0–40.0%) higher than those who did not (12.4, 95% CI: 10.0–14.7%).

The level of pre-diabetes in TB patients who were currently smoking was 34.3% (95% CI: 25.2–43.4%) higher than those never smoked (26.3%; 95% CI: 22.4–33.1%). The proportion of prediabetes was also higher among TB patients with a family history of DM, with the difference nearly reaching the statistical significance (*p* = 0.052). (Table 3).

**Table 3:** The prevalence of pre-diabetes and DM among TB patients, stratified by lifestyles and risk factors, in Hanoi, Vietnam, 2016

	Total TB patients evaluated for DM (n)	TB patients with pre-DM (n)	Prevalence of pre-DM (%; 95% CI)	*p* value	TB patients with DM (n)	Prevalence of DM (%; 95% CI)	*p* value
Total	831	241	29.0 (25.9–32.1)		114	13.7 (11.4–16.1)	
Family history of DM				0.052			**< 0.001**
*Yes*	69	13	18.8 (9.4–28.3)		20	29.0 (18.0–40.0)	
*No*	762	228	29.9 (26.7–33.2)		94	12.4 (10.0–14.7)	
Physical activities				0.283			0.299
*Yes*	278	74	26.6 (21.4–31.8)		43	15.5 (11.2–19.7)	
*No*	553	167	30.2 (26.4–34.0)		71	12.8 (10.0–15.6)	
Frequently of physical activities				0.426			0.147
*> 4 times per week*	168	47	28.0 (21.1–34.8)		30	17.9 (12.0–23.7)	
*1–4 times per week*	63	16	25.4 (14.3–36.4)		9	14.3 (5.4–23.1)	
*1–3 times per month*	30	8	26.7 (9.9–43.5)		2	6.7 (0–16.1)	
*< 1 time per month*	17	3	17.6 (0.0–37.9)		2	11.8 (0–28.8)	
Smoker				**0.036**			0.116
*Never smoker*	510	134	26.3 (22.4–30.1)		61	12.0 (9.1–14.8)	
*Ex-smoker*	213	70	32.9 (26.5–39.2)		36	16.9 (11.8–22.0)	
*Smoker*	108	37	34.3 (25.2–43.4)		17	15.7 (8.8–22.7)	
Drinker				0.705			0.454
*Drinker*	374	106	28.3 (23.8–32.9)		55	14.7 (11.1–18.3)	
*Non-drinker*	457	135	29.5 (25.3–33.7)		59	12.9 (9.8–16.0)	
Type of TB				0.219			0.070
*PTB - smear positive*	391	122	31.2 (26.6–35.8)		60	15.3 (11.8–18.9)	
*PTB - smear negative*	271	68	25.1 (19.9–30.3)		40	14.8 (10.5–19.0)	
*EPTB*	169	51	30.2 (23.2–37.2)		14	8.3 (4.1–12.5)	
TB treatment category				0.138			0.815
*New*	713	200	28.1 (24.7–31.4)		97	13.6 (11.1–16.1)	
*Previously treated*	118	41	34.7 (26.0–43.5)		17	14.4 (8.0–20.8)	
HIV status				0.722			0.555
*Positive*	16	4	25.0 (1.2–48.8)		3	18.8 (10.1–40.2)	
*Negative*	815	237	29.1 (1.6–26.0)		111	13.6 (11.3–16.0)	

*TB* Tuberculosis*; PTB* Pulmonary Tuberculosis*; EPTB* Extra Pulmonary Tuberculosis*; DM* Diabetes mellitus*;*

*CI* confidence interval*; SD* Standard deviation*; VND* Vietnam dong*; BM* Body mass index

*p value by Kruskal-Wallis test and Cuzick test were used for the nonparametric data and to test for trend across ordered groups

### Risk factors associated with DM

In the crude analysis, the associated factors for DM among TB patients were people aged ‘40 years or older’, ‘male’ gender, ‘family members with a history of DM’, ‘pulmonary TB cases’ and a ‘BMI > 25’. After adjusting for other factors in a logistic regression model, the associated factors for DM among TB patients found in this study were age ≥ 40 years and a family history of DM. (Table 4).

**Table 4:** Factors associated with DM among TB patients, in Hanoi, Vietnam, 2016 (*n* = 831)

Characteristics	OR (95% CI)	*p* value	aOR (95% CI)*	*p* value
Age (years)				
< 40	Reference			
40–64	3.56 (2.08–6.07)	< 0.001	6.09 (2.81–13.2)	< 0.001
≥ 65	3.42 (1.88–6.23)	< 0.001	2.65 (1.65–4.25)	< 0.001
Sex				
Female	Reference			
Male	1.74 (1.15–2.63)	0.007	1.62 (0.96–2.75)	0.071
Family history of DM				
No	Reference			
Yes	2.35 (1.55–3.56)	< 0.001	2.71 (1.33–5.50)	0.006
Type of TB				
Extra-pulmonary	Reference			
Smear negative	1.78 (1.00–3.17)	0.044	1.26 (0.83–1.91)	0.272
Smear positive	1.85 (1.07–3.22)	0.024	1.83 (0.86–3.93)	0.119
Smoker				
Never smoke	Reference			
Current smoker	1.32 (0.80–2.16)	0.283	0.75 (0.33–1.71)	0.494
Ex-smoker	1.41 (0.97–2.07)	0.076	0.58 (0.31–1.10)	0.094
BMI				
< 18.5	Reference			
18.5–24.9	1.01 (0.70–1.46)	0.945	1.03 (0.65–1.64)	0.885
> =25	2.22 (1.11–4.42)	0.032	2.27 (0.75–6.82)	0.146
Education				
Primary or lower	Reference			
Secondary; high school	1.09 (0.68–1.74)	0.712	1.07 (0.55–2.05)	0.850
Colleagues/University or higher	0.86 (0.47–1.58)	0.632	0.47 (0.12–1.84)	0.281

*TB* Tuberculosis*; DM* Diabetes mellitus*; OR* Odds ratio*; aOR* Adjusted odds ratio*; CI* Confidence interval*; BMI* Body mass index

*Adjusted for age; sex; residence; education; occupation; marital status; type of TB; family history of DM and BMI in Multivariate logistic regression

### Additional yield of DM

Of the 831 screened TB patients, 114 (13.7%; 95% CI: 11.4–16.1%)) were found to have DM, of those, 50 (44%) were newly diagnosed as DM cases. The number of TB patients needed to be screened to diagnose a new DM case was 17, and the number of TB patients needed to carried-out blood testing to diagnose a new DM case was approximately 16. (Table 5).

**Table 5:** Number of patients needed to screen to find a new DM case among TB patients, in Hanoi, Vietnam, 2016. (*n* = 831)

Characteristic	Total TB patients evaluated for DM (n)	Number of patients with previous DM (n)	Number of newly diagnosed DM patients (n)	Additional yield (%)	Number needed to screen to diagnose a new case (n)
Total	831	64	50	43.9	17
Sex					
*Male*	549	49	39	44.3	14
*Female*	282	15	11	42.3	26
Age, years					
*< 40*	289	4	11	73.3	26
*40–65*	390	45	27	37.5	14
*≥ 65*	152	15	12	44.4	13
Family history of DM					
*Yes*	69	17	3	15.0	23
*No*	762	47	47	50.0	16
Type of TB					
*PTB - smear positive*	391	40	20	33.3	20
*PTB - smear negative*	271	21	19	47.5	14
*EPTB*	169	3	11	78.6	15
BMI, kg/m2					
*Underweight, < 18.5*	289	19	19	50.0	15
*Normal, 18.5–24.9*	518	38	31	44.9	17
*Overweight, > = 25*	24	7	0	0.0	

*TB* Tuberculosis*; PTB* Pulmonary Tuberculosis*; EPTB* Extra Pulmonary Tuberculosis*; DM* Diabetes mellitus*; BMI* Body mass index

## Discussion

The prevalence of DM among TB patients in this study was 13.7% (95% CI: 11.4–16.1%), which is much higher than the estimated prevalence of DM among people aged 18–99 in the general population of Vietnam in 2016 (5.34%; 95% CI: 4.32–7.32%) [13]. The prevalence of DM amongst TB patients is also higher than in the previous study in 2006–2008, in Hanoi, Vietnam (8.8%) [9].

This study showed a 29% prevalence rate of prediabetes amongst TB cases in Hanoi. The prevalence of prediabetes among TB cases was slightly higher than study findings from India (24.5%) [14]; and much higher than study findings in Ethiopia (11.5%) [8]; China (7.8%) [15]; India (7%; 8.5%) [5, 16]. This finding may indicate an increased relatively higher prevalence of DM among TB patients in the future in Vietnam.

The prevalence of DM among TB patients in this study (13.7%) is slightly lower than the global median DM prevalence among TB patients, estimated at 16% (IQR 9.0–25.3%) found by Mahteme et.al [17]. This systematic review had analysed 78 studies reporting DM prevalence among TB patients, representing 33 countries globally. The prevalence of DM amongst TB patients ranged from 1.9% in Cotonou-Benin to 45% in the Ebeye-Mashall Islands [18, 19]. Our study results are comparable with the overall median prevalence of DM among TB patients in Asia, estimated to be 17% (IQR 11.4–25.8%). The prevalence of DM among TB patients in Asia ranges from 5.1% in Salury-South India to 44% in Kerala-India. [5, 17, 20].

The associated risk factors for TBDM comorbidity including sex, age, family history of DM, pulmonary form of TB and positive sputum smear were found in many other studies [17]. In this study, all the above-mentioned factors were found to be associated factors in the bivariate analysis. However, after multivariate analysis, adjusted for other variables, only increasing age and family history of DM remained significant for factors associated with TBDM comorbidity.

Our study found that age was a significant risk factors for TB and DM comorbidity, a finding consistent with many other studies [8, 14, 17, 20, 21]. In our study, the risk of having DM was 6 times higher in the 40–65 age group when compared to those patients under 40 years old; the risk dropped to approximately 3 times higher in the age group 65 years old and older when compare to age groups of patients less than 40 years old. This may be explained by the link between increasing age and decreases in immune status, one risk factor for both TB and DM [8].

Having a family history of DM is known as a risk factor for DM [7, 12]. Our study also found the risk of DM is 2.7 times higher among TB patients who had a family history of DM. This finding was consistent with other studies conducted in Ethiopia, China, and India [8, 14, 17, 21].

Many studies have also reported a higher prevalence of DM and prevalence of prediabetes amongst males compared to females [6, 20]. In our study, male TB patients were also identified to have a higher risk of DM in the bivariate analysis. One reasons for this may be a higher frequency of habits such as smoking and drinking alcohol among men. However, the multivariate analysis could not show a significant association.

In this study, 17 TB cases were needed to be screened in order to diagnose one new DM case. This number is high compared to other studies in Ethiopia (20 cases) [8]; Gujarat-India (25 cases) [16]; and South, India (31 cases) [5]. Such a finding suggests that the implementation of a DM screening strategy for TB patients in Hanoi could be high-value.

This study also demonstrated that a high level of participation was possible for DM testing amongst TB patients in Hanoi, Vietnam. In 2011, in the collaboration framework, WHO and the UNION have recommended screening for DM among TB patients [3]. Given the high prevalence of DM, especially the high prevalence of prediabetes among TB patients, Vietnam NTP should collaborate with diabetes programme and establish a systematic screening process for DM among TB patients. This program would enable early diagnosis of DM to be made and reduce morbidity and mortality amongst TB patients.

This study has some limitations. As the study was conducted in an urban area, Hanoi city, the findings may not be representative of the entire country, particularly rural areas. Secondly, the study was only implemented among TB patients who were diagnosed and treated in NTP system, thus it may not be representative of TB patients diagnosed and treated in the private or non-NTP public sectors, or TB patients who have not been diagnosed. Thirdly, decisions on the diagnosis of DM were based on FBG only, this testing methodology may be less sensitive than glycosylated haemoglobin or oral glucose tolerance tests. Finally, this study used the standard cut-off points for BMI categories, it did not use cut-off points specific to Asian populations as recommended by the WHO.

## Conclusions

In conclusion, this study illustrated the high prevalence of DM and prediabetes among TB patients in Hanoi, Vietnam. This was especially true for persons aged over 40 years old or who had a family history of DM. Given the high prevalence of DM, particularly the high prevalence of prediabetes among TB patients, this study recommends the Vietnam NTP need to collaborate with diabetes programme to establish a systematic screening process for DM amongst TB patients.

## Abbreviations

ADA: The American Diabetes Association
BMI: Body Mass Index
CI: Confidence interval
DM: Diabetes Mellitus
EPTB: Extra Pulmonary Tuberculosis
FBG: Fasting blood glucose
IDF: International Diabetes Federation
IFG: Impaired fasting glucose
IQR: Interquartile range
NTP: National Tuberculosis Programme
OR: Odds Ratio
PTB: Pulmonary Tuberculosis;
SD: Standard Deviation
TB: Tuberculosis
UNION: The International Union Against Tuberculosis and Lung Disease
VND: Vietnam Dong
WHO: World Health Organization
